# The Influence of Declining Air Lead Levels on Blood Lead–Air Lead Slope Factors in Children

**DOI:** 10.1289/ehp.1307072

**Published:** 2014-03-25

**Authors:** Jennifer Richmond-Bryant, Qingyu Meng, Allen Davis, Jonathan Cohen, Shou-En Lu, David Svendsgaard, James S. Brown, Lauren Tuttle, Heidi Hubbard, Joann Rice, Ellen Kirrane, Lisa C. Vinikoor-Imler, Dennis Kotchmar, Erin P. Hines, Mary Ross

**Affiliations:** 1 National Center for Environmental Assessment, U.S. Environmental Protection Agency, Research Triangle Park, North Carolina, USA; 2 School of Public Health, Rutgers University, Piscataway, New Jersey, USA; 3 ICF International, Fairfax, Virginia USA; 4 School of Architecture, The University of Texas at Austin, Austin, Texas, USA; 5 Office of Air Quality Planning and Standards, U.S. Environmental Protection Agency, Research Triangle Park, North Carolina, USA

## Abstract

Background: It is difficult to discern the proportion of blood lead (PbB) attributable to ambient air lead (PbA), given the multitude of lead (Pb) sources and pathways of exposure. The PbB–PbA relationship has previously been evaluated across populations. This relationship was a central consideration in the 2008 review of the Pb national ambient air quality standards.

Objectives: The objectives of this study were to evaluate the relationship between PbB and PbA concentrations among children nationwide for recent years and to compare the relationship with those obtained from other studies in the literature.

Methods: We merged participant-level data for PbB from the National Health and Nutrition Examination Survey (NHANES) III (1988–1994) and NHANES 9908 (1999–2008) with PbA data from the U.S. Environmental Protection Agency. We applied mixed-effects models, and we computed slope factor, d[PbB]/d[PbA] or the change in PbB per unit change in PbA, from the model results to assess the relationship between PbB and PbA.

Results: Comparing the NHANES regression results with those from the literature shows that slope factor increased with decreasing PbA among children 0–11 years of age.

Conclusion: These findings suggest that a larger relative public health benefit may be derived among children from decreases in PbA at low PbA exposures. Simultaneous declines in Pb from other sources, changes in PbA sampling uncertainties over time largely related to changes in the size distribution of Pb-bearing particulate matter, and limitations regarding sampling size and exposure error may contribute to the variability in slope factor observed across peer-reviewed studies.

Citation: Richmond-Bryant J, Meng Q, Davis A, Cohen J, Lu SE, Svendsgaard D, Brown JS, Tuttle L, Hubbard H, Rice J, Kirrane E, Vinikoor-Imler LC, Kotchmar D, Hines EP, Ross M. 2014. The Influence of declining air lead levels on blood lead–air lead slope factors in children. Environ Health Perspect 122:754–760; http://dx.doi.org/10.1289/ehp.1307072

## Introduction

Lead (Pb) in blood (PbB) reflects all sources and pathways of human exposure to Pb; hence, dramatic declines in PbB levels reported in the National Health and Nutrition Examination Survey (NHANES) have coincided with the phaseout of leaded gasoline and other regulatory restrictions on Pb in industry and consumer products (Brody et al. 1994; Pirkle et al. 1994, 1998). Current sources of Pb potentially contributing to Pb body burden include ambient sources such as some piston-engine aircraft fuel and Pb-acid battery recycling operations, whereas nonambient sources include Pb in building materials used in older homes [U.S. Environmental Protection Agency (EPA) 2011]. Given the multitude of potential Pb sources and pathways of exposure, it is often difficult to distinguish the proportion of PbB attributed to air-related pathways—those pathways where Pb passes through ambient air from a source to human exposure. Air-related pathways include inhalation of airborne Pb (freshly emitted or resuspended) and ingestion of Pb that, once airborne, has made its way into indoor dust, outdoor dust or soil, dietary items, and drinking water.

Epidemiologic studies have explored the relationship between PbB and ambient air Pb (PbA) with regression or other statistical modeling techniques using individual or aggregated data (e.g., Brunekreef 1984; Hayes et al. 1994; Schwartz and Pitcher 1989; Tripathi et al. 2001). Statistical models typically have PbB as the dependent variable, and PbA is either the only independent variable or is adjusted by one or more covariates, such as race/ethnicity or home age. Model formulations have varied with respect to use of transformation functions (e.g., applying a logarithm to terms on one or both sides of the equation) and stratification of the sample population. As a result, point-wise estimation of the slope factor, or the change in PbB per unit change in PbA—d[PbB]/d[PbA]—has also varied between and within statistical models; in the latter case, within-model variation occurs for nonlinear formulations.

The overall objective of this work is to evaluate how the relationship between PbB levels in children and PbA levels has changed following the phaseout of leaded gasoline and tightened controls on environmental Pb emissions over the past 30 years. In this study we explored the relationship of PbB with PbA over the course of several papers that describe the statistical modeling techniques and results for effect estimates for PbA, calculate slope factor for different PbA size fractions, and investigate effect modification of the PbB–PbA relationship. The results presented here focus on the comparison of the slope factor results for PbA concentration data in total suspended particles (TSP) with results from studies in the literature performed between 1970 and 2011.

## Methods

*Data sets*. Richmond-Bryant et al. (2013) provided a detailed description of the data sets used in this analysis, which is summarized briefly here. We obtained participant-level data for this analysis from the NHANES III (1988–1994) and Continuous NHANES (1999–2008) (hereafter referred to as NHANES 9908) surveys [Centers for Disease Control and Prevention (CDC) 2014]. We obtained the NHANES data in 2-year cycles. We also used the data imputation scheme described by Richmond-Bryant et al. (2013) for this data set to account for missing data, except for the PbB variable. When PbB was missing, we omitted the data record because it was the dependent variable. We stratified the data by age groups used in NHANES (1–5 years, 6–11 years) to reflect potential differences in Pb storage, Pb distribution, Pb metabolism, and hand-to-mouth behavior among young and older children. However, we did not use other covariates in the analysis because the PbA thereby accounted for all air-related pathways contributing to PbB (U.S. EPA 2013).

Air Quality System (AQS) data variables included TSP in PbA (TSP-PbA) concentration, monitor identification number, latitude, longitude, and date. We used particulate matter (PM) sampled as TSP from a high-volume sampler for this analysis because other peer-reviewed studies, to which these results were compared, employed TSP-PbA. Roughly 270 TSP monitors for sampling PbA were active as of February 2010 (U.S. EPA 2008a). Over the course of NHANES 9908, 456 monitor locations corresponded to PbB data and collected 108,126 data points. During NHANES III, 756 monitor locations corresponded to PbB data and collected 178,036 data points. Samples were collected on a 24-hr basis. Among those monitors reporting data acquisition frequency, 1.9% reported collected data each day, 0.2% every other day, 3.7% every third day, 83.5% every sixth day, 10.6% every twelfth day, and 0.2% every 30 days. On the basis of an anticipated 286,162 samples for the years of sampling at the frequencies listed above, we estimated 64% of 24-hr data to be missing for NHANES 9908 and 26% of 24-hr data to be missing for NHANES III. To be included in this study, we required 75% data completeness for monitors for the years in which the monitors were active. However, many of the TSP-PbA monitors were not active for all years coinciding with NHANES III and NHANES 9908. We averaged PbA concentrations over 365 days before the PbB sample. We did not use PbA observations with missing and zero values of concentration in the analyses because the statistical model described below used a logarithmic transformation. However, we did not omit non-zero data below the method detection limit (MDL) for sampling and analysis of PbA, to minimize upward bias of the PbA data distribution. MDL was ≤ 0.002 μg/m^3^ for 60% of the monitors. MDL was ≤ 0.01 μg/m^3^ for 98% of the monitors, and for one monitor, MDL was 0.05 μg/m^3^. For NHANES 9908, 23% of data averaged over 365 days were below MDL, and 3.8% of the data averaged over 365 days had zero values and hence were omitted. For NHANES III, 6.2% of the data averaged over 365 days were below MDL, and 1.5% of the data averaged over 365 days had zero values. Because the data were averaged over 365 days, we deemed data imputation unnecessary. However, we acknowledge that omission of data would induce uncertainty in the PbA distribution.

Data for PbA and PbB were merged into one database based on the NHANES participants’ location and date of examination. Staff at the National Center for Health Statistics Research Data Center (RDC) (CDC 2012) merged the data to maintain confidentiality of the NHANES participants’ personal data. When a TSP-PbA monitor was located within 4 km of a census block–group centroid in which an NHANES participant resided and a 24-hr TSP-PbA concentration measurement fell within 7 days of the NHANES participant examination date, RDC staff assigned the 365-day average TSP-PbA concentration from that TSP-Pb sampler to that participant. We applied a 7-day window for the data merger because the TSP-PbA data were typically obtained every 6 days.

We selected the 4-km buffer size based on the Code of Federal Regulations (U.S. EPA 1979a), which established microscale (100 m), middle-scale (100–500 m), neighborhood-scale (within 4 km), urban-scale (10s of km), and regional-scale (100s of km) measurements all as potential buffers in which PbA concentration could be measured. Neighborhood scale is cited as representative of PbA concentrations in urban areas not near sources (U.S. EPA 1979b). Although the 4-km neighborhood scale is somewhat arbitrary, it is well known that larger Pb particles tend to settle out, whereas smaller particles have the potential for longer transport (U.S. EPA 1979a).

After the data were merged, RDC staff deidentified the data set by removing geographic location and date fields before use of the database for analysis. The University of North Carolina Institutional Review Board (IRB) reviewed the study design and deemed it exempt from IRB oversight. Richmond-Bryant et al. (2013) detailed the data merge procedures briefly described here.

*Statistical analysis*. We employed a simplified univariate approach for this analysis to estimate the change in PbB in response to a change in PbA. Use of a univariate model enabled all air-related pathways of Pb exposure to be incorporated into the one term for PbA, because covariates such as housing age could reflect exposure to deposited PbA. The detailed multilevel linear mixed-effects (LME) modeling approach is presented by Richmond-Bryant et al. (2013). PbA was a census block group–level fixed effect in the model. The random effects at the census block group level were random intercepts for each census block group identification number, and the residual error terms were random effects at the individual level. We treated subject age strata and NHANES survey (NHANES III or NHANES 9908) as stratification variables. We treated the geographical location as a random effect. The LME model was of the form

ln[*PbB_i,j_*] = β_0_ + *b_j_* + β*_PbA_*ln[*PbA_j_*] + ε*_i,j_*, [1]

where *PbB_i,j_* is the PbB for the *i*th individual living in the *j*th census block group, [*PbA_j_*] is the average PbA concentration obtained at the *j*th census block group, and β*_PbA_* is the slope of ln(*PbB*) on ln(*PbA*) for the *j*th census block group (and ln denotes the natural logarithm). β*_0_* is the overall intercept, *b_j_* is a census block group–level random normal intercept with mean zero and variance τ^2^, and ε*_i,j_* is a random normal variable with mean zero and variance σ^2^. We performed likelihood ratio testing to test the statistical significance of ln(*PbA*) by running Equation 1 with and without the PbA variable. Because this model does not include demographic and other covariates, their potential effects on PbB are incorporated in the random census block–group effect and residual error terms. The model used by Richmond-Bryant et al. (2013) adds various demographic and covariate fixed-effect terms to the righthand side of Equation 1. We used simulations to evaluate the choice of the ln–ln model to fit the data, and we described the simulations in the Supplemental Material, “Simulation methods,” pp. 2–4, and Tables S1 and S2. We evaluated the simulated model results against each other and against the results of the model using the merged data set. We selected the ln–ln model fit to the simulated data as most similar to the model fit for the merged data sets. The RDC maintains a strict policy of prohibiting restricted-access NHANES data users to plot real data points with the regression curve or to output residuals, to avoid the possibility that an individual participant’s identity could be reconstructed by using their exposures as measured at the nearest monitor to find the participant’s approximate location and then to match the associated PbB level and covariates.

Rabe-Hesketh and Skrondal (2006) have found that incorporation of survey weights in multilevel models using a pseudo-likelihood approach can lead to biased estimates if the level-1 weights are different from 1 and if the cluster sizes are small. For this reason, we did not apply sampling weights calculated for NHANES to adjust for oversampling segments of the population (e.g., for race/ethnicity, age group, or urbanization) in the LME analysis.

*Computation of slope of PbB to PbA*. We employed Equation 1 for computing the slope factors used to interpret the relationship between PbB and PbA. For this study, we derived an instantaneous slope factor, d[*PbB*]/d[*PbA*], for the ln–ln model in lieu of interval-based approximations for slope used in earlier studies (e.g., Brunekreef 1984; Hayes et al. 1994):

d[*PbB*]/d[*PbA*] = e^^β^_0_^β*_PbA_*[*PbA*]^^β^^*^_PbA_^*^^–1^^^.^ [2]

In addition to ensuring that the PbA term accounted for all air-related pathways, use of Equation 1 allowed for straightforward calculation of the slope factor because calculation of slope factor with a covariate model would entail testing of several hypothetical scenarios to estimate covariate values. With a combination of categorical and continuous covariates, this step is not straightforward. Brunekreef (1984) recognized that computation of slope factor from a model not including covariates may result in some amplification of slope factor. We assumed that the average *b_j_* over all census block groups was equal to zero, so the random intercept does not appear in Equation 2.

*Literature review*. We included studies computing slope factor that were presented by U.S. EPA (2013) for comparison with the NHANES analyses (Brunekreef 1984; Hayes et al. 1994; Hilts 2003; Schwartz and Pitcher 1989; Tripathi et al. 2001). To ensure that we did not miss additional relevant studies, we also conducted a Web of Science (http://thomsonreuters.com/thomson-reuters-web-of-science/) search using the topic queries “blood Pb” and “air Pb.” We retrieved 162 articles from this search. We reviewed the titles and abstracts from these references to determine if the associations between PbB and PbA were studied in those articles. We reduced the pool of articles to 11 after the title–abstract review. We reviewed the 11 remaining articles in full, and only three new articles—Bierkens et al. (2011), Ranft et al. (2008), and Zahran et al. (2013)—included a statistical model of PbB as a function of PbA. For each of the statistical models presented in these eight studies, we computed slope factor based on Equation 2. If any study used base-10 logarithms in lieu of natural logarithms, then we replaced the exponential with a base of 10 in Equation 2.

## Results and Discussion

**Figure 1:**
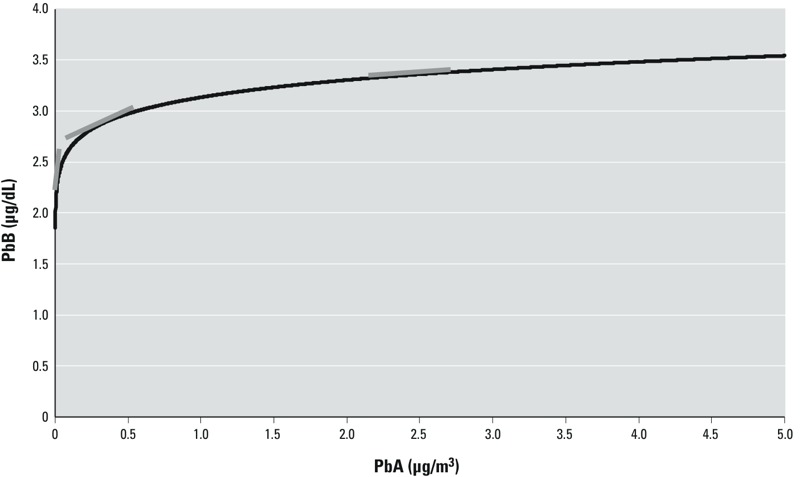
Calculated PbB levels for a range of PbA concentrations for the NHANES 9908 models for children 1–5 years of age. Tangent lines are shown in gray to illustrate how slope factor decreases with increasing PbA along the ln–ln model.

Regression results show that β*_PbA_* decreased substantially between NHANES III and NHANES 9908, as shown in Table 1. For NHANES III, β*_PbA_* was 0.140 *(p* = 0.011) for the 1- to 5-year age group and 0.154 *(p* = 0.067) for the 6- to 11-year age group. For NHANES 9908, β*_PbA_* was 0.076 *(p* = 0.12) for the 1- to 5-year age group and 0.155 *(p* = 0.0012) for the 6- to 11-year age group. The magnitude of β*_PbA_* for Equation 1 of NHANES 9908 was 54% and 101% of that for NHANES III for the 1- to 5-year and 6- to 11-year age groups, respectively. Likelihood testing produced the same findings with respect to statistical significance of ln(PbA) for both NHANES cycles and models. Figure 1 displays the modeled relationship between PbB and PbA for children 1–5 years of age based on the NHANES 9908 data. The ln–ln model design is evident from the steepness of the tangent slope factor for small PbA with a sharp decrease in slope factor with increasing PbA.

**Table 1:** Regression coefficients (β ± SE) for merged PbB–PbA data set, based on annual average TSP‑Pb data.

Variable	NHANES 9908	NHANES III
1–5 years
Residual	0.286 ± 0.0370^#^	0.473 ± 0.031^#^
Intercept	1.142 ± 0.219^#^	1.932 ± 0.181^#^
ln(PbA)	0.076 ± 0.0480	0.140 ± 0.054*
Variance (location random effect)	0.138^#^	0.105**
6–11 years
Residual	0.228 ± 0.027^#^	0.310 ± 0.036^#^
Intercept	1.171 ± 0.203^#^	1.812 ± 0.279^#^
ln(PbA)	0.155 ± 0.046**	0.154 ± 0.083
Variance (location random effect)	0.184^#^	0.349^#^
^#^0.01 < *p* ≤ 0.05. **0.001 < *p* ≤ 0.01. *p ≤ 0.001.		

We observed little difference among the slope factors between NHANES III and NHANES 9908; this information is shown in Table 2 at the median concurrent PbA concentration. For NHANES III, slope factor was 16.4 μg/dL per μg/m^3^ for the 1- to 5-year age group and 15.7 μg/dL per μg/m^3^ for the 6- to 11-year age group at a median PbA of 0.036 μg/m^3^ and 0.037 μg/m^3^, respectively; slope factor was statistically significantly greater than zero. For NHANES 9908, slope factor was also statistically significantly greater than zero. For the 1- to 5-year and 6- to 11-year groups, slope factor was 15.3 and 16.5 μg/dL per μg/m^3^ at PbA of 0.011 to 0.016 μg/m^3^, respectively; these slope factor values were close to values calculated from the NHANES III simulations. Similarities in slope factor for the 1- to 5-year and 6- to 11-year age groups arose because differences between the models’ β*_PbA_* and β_0_ among the NHANES survey cycles were offset by the reduction in PbA from 0.036 to 0.037 μg/m^3^ for the 1988–1994 survey down to 0.011 to 0.016 μg/m^3^ for the 1999–2008 survey. Given the complex structure of Equation 2, slope factor increases with decreasing PbA when β*_PbA_* and β_0_ are fixed. Larger values for β_0_ and β*_PbA_* will also result in higher slope factor. β_0_ and β*_PbA_* for NHANES III were > 1.75 times higher than for NHANES 9908 in the 1- to 5-year age group, and β_0_ was roughly 1.5 times higher in the 6- to 11-year group for NHANES III compared with NHANES 9908.

**Table 2:** Comparison of studies computing slope factor at median PbA for each study.

Reference	Adjustment for confounders	Population	*n*	Years	Air metric (source)	PbA (μg/m^3^)	Model form	Slope factor ± SE
Schwartz and Pitcher 1989	Age, race, sex, income, degree of urbanization, region of country, nutrition intake, head of household’s education level, smoking, alcohol, occupational exposure	0.5–74 years (white, stated to be similar to total population), United States	9,987	1976–1980	Multiyear (1976–1980); units (μg/dL per 100 metric tons gasoline Pb/day) (U.S. Dept. of Energy and U.S. EPA estimates of gasoline usage, 0.23 μg/m^3^ per 100 metric tons gasoline Pb/day)	Median: 0.98 Range: 0.59–1.3	Linear	9.30 ± 0.192
Present study	No	1–5 years, United States	654	1988–1994	Annual average (AQS)	Median: 0.037 5%–95%: 0.013–0.11	Ln–ln	16.4 ± 4.54
Present study	No	1–5 years, United States	205	1999–2008	Annual average (AQS)	Median: 0.011 5%–95%: 0.0016–0.093	Ln–ln	15.3 ± 7.24
Schwartz and Pitcher 1989	Age, race, sex, income, degree of urbanization, region of country, nutrition intake, head of household’s education level, smoking, alcohol, occupational exposure	0–5 years (black, stated to be similar to total population), Chicago, IL	5,476	1976–1980	Multiyear (1976–1980); units (μg/dL per 100 metric tons gasoline Pb/day) (U.S. Dept. of Energy and U.S. EPA estimates of gasoline usage, 0.23 μg/m^3^ per 100 metric tons gasoline Pb/day)	Median: 0.89 Range: 0.36–1.3	Linear	8.57 ± 0.728
Brunekreef 1984 (meta-analysis)	No	Children varying age ranges, various locations	> 190,000	1970–1982 (years missing from 2 studies)	Not always stated (references within Brunekreef 1984)	Median: 2.0 Range: 0.0–24	Ln–ln	3.85
Brunekreef 1984 (meta-analysis)	No	Children with pbb ≤ 20 μg/dL	> 190,000	1970–1982 (years missing from 2 studies)	Not always stated (references within Brunekreef 1984)	Median: 2.0 Range: 0.0–24	Ln–ln	1.72
Hayes et al. 1994	No	0.5–6 years, Chicago, IL	9,604	1968–1988	Quarterly average (Illinois Environmental Protection Agency)	Median: 0.37 Range: 0.054–1.17	Ln–ln	12.2
Hilts 2003	No	0.5–6 years, Trail, British Columbia	220–460 (estimated)	1991–2000	Quarterly average (operated by the Smelter Company)	Range of GM: 0.03–1.1	Linear	6
Bierkens et al. 2011	No	< 6 years, European Union members	22	1981–2008	Annual average (European Environmental Agency)	Median: 0.098 Range: 0.0010–3.0	Log-log	2.83
Zahran et al. 2013	Pb facility, capillary blood draw, sex, year, meteorology	0 years, Detroit, MI	19,265	2001–2009	Monthly average (estimated by model)	Mean: 0.004 Median: 0.004 SD: 0.001	Ln–ln	34.0 ± 1.27
Zahran et al. 2013	Pb facility, capillary blood draw, sex, year, meteorology	1 years	76,070	2001–2009	Monthly average (estimated by model)	Mean: 0.004 Median: 0.004 SD: 0.001	Ln–ln	57.3 ± 1.12
Zahran et al. 2013	Pb facility, capillary blood draw, sex, year, meteorology	2 years	58,500	2001–2009	Monthly average (estimated by model)	Mean: 0.004 Median: 0.004 SD: 0.001	Ln–ln	62.5 ± 1.77
Zahran et al. 2013	Pb facility, capillary blood draw, sex, year, meteorology	3 years	66,507	2001–2009	Monthly average (estimated by model)	Mean: 0.004 Median: 0.004 SD: 0.001	Ln–ln	37.2 ± 2.20
Zahran et al. 2013	Pb facility, capillary blood draw, sex, year, meteorology	4 years	67,061	2001–2009	Monthly average (estimated by model)	Mean: 0.004 Median: 0.004 SD: 0.001	Ln–ln	30.9 ± 2.08
Zahran et al. 2013	Pb facility, capillary blood draw, sex, year, meteorology	5 years	34,073	2001–2009	Monthly average (estimated by model)	Mean: 0.004 Median: 0.004 SD: 0.001	Ln–ln	35.5 ± 2.55
Zahran et al. 2013	Pb facility, capillary blood draw, sex, year, meteorology	6 years	18,911	2001–2009	Monthly average (estimated by model)	Mean: 0.004 Median: 0.004 SD: 0.001	Ln–ln	24.8 ± 3.33
Zahran et al. 2013	Pb facility, capillary blood draw, sex, year, meteorology	7 years	8,649	2001–2009	Monthly average (estimated by model)	Mean: 0.004 Median: 0.004 SD: 0.001	Ln–ln	21.3 ± 4.94
Zahran et al. 2013	Pb facility, capillary blood draw, sex, year, meteorology	8–10 years	13,610	2001–2009	Monthly average (estimated by model)	Mean: 0.004 Median: 0.004 SD: 0.001	Ln–ln	16.7 ± 3.81
Present study	No	6–11 years, United States	272	1988–1994	Annual average (AQS)	Median: 0.036 5%–95%: 0.015–0.11	Ln–ln	15.7 ± 6.03
Present study	No	6–11 years, United States	204	1999–2008	Annual average (AQS)	Median: 0.016 5%–95%: 0.0021–0.059	Ln–ln	16.5 ± 3.77
Tripathi et al. 2001	No	6–10 years, Mumbai, India	544	1984–1996	Multiyear (1984–1996)	Median of GM: 0.37 Range of GM: 0.10–41	Linear	3.62
Ranft et al. 2008	Soil, sex, age, nationality of child, parents’ education, exposure to ETS	6–11 years, Duisburg, Germany	843	1983–2000	Annual average	Median: 0.1 Range: 0.025–0.729	Ln-linear	3.19
ETS, environmental tobacco smoke.								

We computed slope factors presented in the above paragraph with the fixed-effects slopes derived from the mixed models (Table 2). Additionally, we added a random effect to the intercept to account for the influence of geography on an individual’s slope factor in the LME model. Given that we added the random effect to the intercept, it would have the effect of multiplying slope factor by exp(*b_j_*), such that Equation 2 would become

d[*PbB*]/d[*PbA*] = e^^β^_0_^*^+^b^_j_^*β*_PbA_*[*PbA*]^^β^^*^_PbA_^*^^–1^^^.^ [3]

The variance of the random effect, given in Table 1, was used to calculate how the slope factor would change over the 95% confidence interval of the random effect, by adding ±1.96[var(*b_j_*)/*n*]^0.5^ in place of *b_j_* in Equation 3. This results in the slope factor being multiplied by a factor within the interval [0.93,1.07] to capture the influence of the random effect across the age groups and survey cycles.

We compared slope factors from the results of this study with those computed from related studies in the literature. Although many studies provided slope factor, we recomputed it here as the first derivative of the PbB–PbA regression relationship presented in each study to ensure that the computational approach was equivalent because some studies estimated slope factor based on interval approximation (e.g., Brunekreef 1984; Hayes et al. 1994). Additionally, we computed slope factor using the median PbA to maintain consistency among the models. For the ln–ln models, use of low current levels of PbA (e.g., for NHANES 9908, median PbA ranged from 0.011 to 0.016 μg/m^3^) would drive slope factor upward compared with using PbA closer to 1 μg/m^3^ for older studies, because the ln–ln curves are steeper at lower PbA; this is evident in Figure 1. Linear models had constant slope factor, so that the relative difference in slope factor between the ln–ln and linear models increased with decreasing PbA.

Most studies of the PbB–PbA relationship employed data from the period when Pb was used as an antiknock agent in gasoline and a more prevalent raw material in manufactured goods in the United States and abroad (Brunekreef 1984; Hayes et al. 1994; Hilts 2003; Ranft et al. 2008; Schwartz and Pitcher 1989; Tripathi et al. 2001). We summarized these studies in Table 2 along with data from this study. We observed variability in the studies’ median slope factor estimates; Brunekreef (1984), in a meta-analysis of earlier studies associating PbB with PbA, also noted such variability. For the studies that obtained most data when industrial and automotive Pb usage was higher, slope factor ranged from 3.2 to 12.2 (Brunekreef 1984; Hayes et al. 1994; Hilts 2003; Ranft et al. 2008; Schwartz and Pitcher 1989; Tripathi et al. 2001). Bierkens et al.’s (2011) study of PbA exposure throughout the European Union (EU) included many data from after 2000, when EU countries had phased out Pb from automotive gasoline and saw diminished industrial use. They observed a slope factor of 2.8 μg/dL per μg/m^3^ among children 0–6 years of age. Likewise, Zahran et al.’s (2013) study of PbA exposure in Detroit, Michigan, used data from 2001–2009 and observed slope factor ranging from 17 to 63 μg/dL per microgram per cubic meter for children 0–10 years of age in 1- to 3-year age groupings. The U.S. EPA (2007) reviewed the evidence before 2007 and determined that slope factor overall was between 3 and 10 μg/dL per μg/m^3^ (i.e., an increase in PbA concentration of 1 μg/m^3^ would translate to increases in PbB levels of 3–10 μg/dL).

Table 2 shows comparison of slope factor, computed at the studies’ median PbA among this and other studies modeling PbB as a function of PbA. All computed slope factors were statistically significant. Median slope factor across the 21 models presented in Table 2 for children 0–11 years of age was 16.4 μg/dL per μg/m^3^. Slope factors across the studies had an interquartile range of 24.9 μg/dL per μg/m^3^. Computations from the NHANES III and NHANES 9908 analyses and for Zahran et al. (2013) yielded higher slope factor than the other studies modeling PbB vs. PbA.

**Figure 2:**
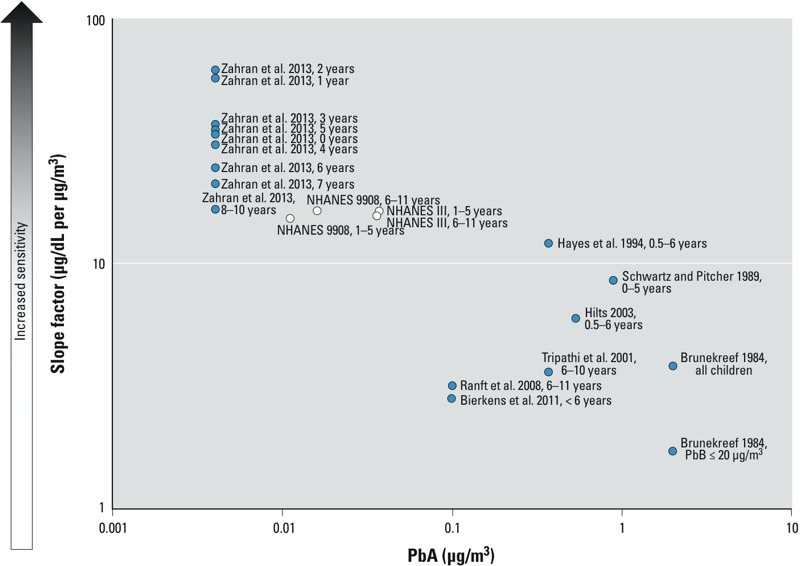
Slope factor versus PbA for children 0–11 years of age, with slope factor computed for the median PbA at the time of the study. Data for the present study are shown with white circles.

**Figure 3:**
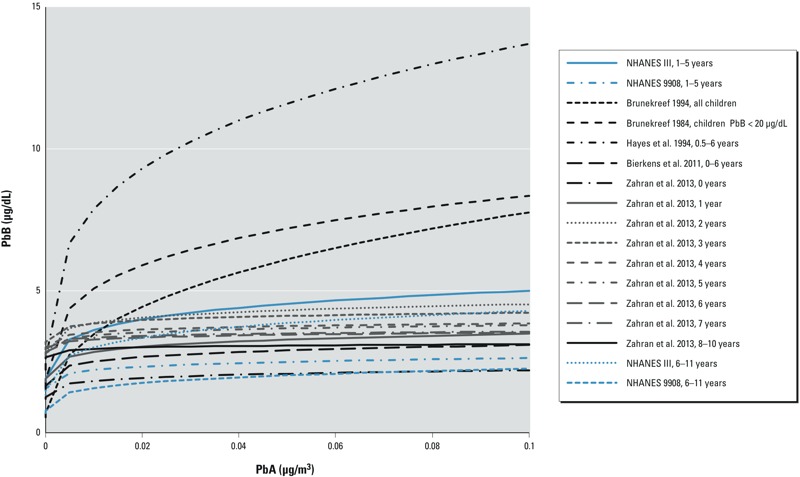
Calculated PbB levels for a range of PbA concentrations for all studies with study participants 0–11 years of age and using a ln–ln or log–log model of the PbB–PbA relationship.

To explore potential reasons for higher slope factor values among children 0–11 years of age for the NHANES analyses and for Zahran et al. (2013) compared with the other studies in the literature, slope factor was plotted against median PbA for these studies (Figure 2). Here, the median PbA values (0.011–0.037 μg/m^3^) for the four NHANES analyses and for the Zahran et al. (2013) study were substantially lower than median PbA for the other studies (0.098–2 μg/m^3^). Hence, the study results taken in concert suggest that slope factor increases among children with decreasing exposure to PbA. This is consistent with the curve of PbB versus PbA presented in Figure 1 and with the inverse mathematical dependence of slope factor with PbA, as seen in Equation 2. The declining relationship between slope factor and PbA in ln–ln space supports use of the ln–ln plot to describe the relationship between PbB and PbA. Furthermore, examination of slope factor for many studies across a range of PbA lends additional credence to the notion that slope factor increases with decreasing PbA, as illustrated in Figure 3 for the PbB–PbA models computed from the studies of childhood exposure included here. In this sense, the decreased PbA concentrations for the NHANES and Zahran et al. (2013) studies account for higher slope factor observed in Figure 2. When PbA is already low, additional reduction in PbA would then produce a larger proportional decrease in PbB compared with the same reduction at a higher initial PbA. Variation in the β*_PbA_* and β_0_ of the PbB–PbA curve causes some of these differences. For example, the curves from Brunekreef (1984) and Hayes et al. (1994) have much larger β*_PbA_* compared with the other models. In other cases, the different model curves in Figure 3 illustrate that β*_PbA_* of many of the models is similar, but β_0_ differs. These differences in β_0_ are likely responsible for the amount of scatter observed in Figure 2 among slope factor for higher PbA.

The large amount of scatter in the graph of slope factor versus PbA presented in Figure 2 suggests variation in the form of the ln–ln relationship between PbB and PbA over the years 1970–2009. During this time period, several changes to population Pb exposure have occurred. By 2000, most countries had phased out Pb additives from gasoline, and the timing and duration of the phaseouts varied across the studies examined here. Additionally, environmental Pb emissions have decreased throughout the last 40 years (U.S. EPA 2008b). In a multivariate model using the NHANES III and NHANES 9908 data, the magnitude of ln(PbB) effect estimates varied between the NHANES cycles for socioeconomic and co-exposure factors (Richmond-Bryant et al. 2013). Scatter in slope factor may also reflect differences in the covariates between the survey cycles.

This study is not without limitations. First, to preserve NHANES participant confidentiality, the RDC restricted printing or plotting the study data or residuals once the NHANES and AQS data sets were merged. As a result, visual inspection of the fit of the regression curves was not possible. Second, we used the 4-km buffer size around the monitors to maintain the small distances likely travelled by Pb particles without losing excessive statistical power at the monitors. However, we lost some power, because application of the monitoring buffers caused reduction of the NHANES III sample population from 9,368 to 926; similarly, the NHANES 9908 sample population decreased from 8,244 to 409 after applying the buffers. Limiting the overall number of participants reduced the statistical significance of the study results. Stratifying the population by age and survey further reduced sample size. Third, use of the 4-km buffers also potentially caused exposure misclassification, if PbA concentration was sometimes incorrectly assigned to an NHANES participant. We could not test other buffer sizes, because only RDC staff could perform the data merger. Fourth, selecting the centroid of the census block group in which the monitor resides as the comparison metric for the monitor location likely added exposure error because we did not know the proximity between the monitors and the individuals. Fifth, changes in PbA emissions from NHANES III to NHANES 9908 suggest that the size distribution of PbA particles would have changed, since Pb emitted to the air by automobiles were in the fine fraction; a recent review of the PbA literature supports this idea (Cho et al. 2011). This shift would change the amount of bias and uncertainty in PbA measurements, because errors in the PbA monitoring method increase with increasing particle size (Wedding et al. 1977). Additionally, larger particles are more likely to settle from the air compared with smaller particles (Fuchs 1964), so that a smaller fraction of the population may have been exposed to Pb-bearing particles in 1999–2008 compared with 1988–1994. At the same time, the upward shift in size distribution of Pb-bearing particles changes the site of deposition within the respiratory system, potentially increasing the amount of ingested versus inhaled Pb and related absorption (Niu et al. 2010). These two factors could have a real impact on slope factor. Sixth, the literature review within this current study used PbB–PbA data for distinct populations and analyzed by different models. Some of the trend and/or scatter might be attributed to those differences. Last, the results were not amenable to making national inference, because we did not apply the sample weights in the LME, as described in “Methods.” However, the study did sample a large cross-section of the United States. Except for Schwartz and Pitcher (1989), previous studies of the relationship between PbB and PbA in the United States were limited to specific cities. The limitations of the study are balanced by gaining an improved understanding of the PbB–PbA association for the period following the phase out of Pb as an antiknock agent in gasoline based on the linkage of two large databases.

## Conclusions

The primary finding of this cross-sectional epidemiologic study is that the slope factor increases with decreasing PbA for children 0–11 years of age, albeit with a large amount of scatter across the studies reviewed for comparison with the NHANES estimates. Because slope factor is higher at low PbA, larger relative PbB reductions among children may be achieved at low PbA levels. At the same time, we need more studies of the conditions influencing slope factor to interpret the results presented here. Additional analyses exploring the effect of particle size cuts for the PbA measurements and effect modification factors influencing slope factor are in progress.

## Supplemental Material

(227 KB) PDF
