# Supplementary material for: The Influence of Declining Air Lead Levels on Blood Lead–Air Lead Slope Factors in Children
Source: Environ Health Perspect. 2014 Mar 25;122(7):754–60. doi: 10.1289/ehp.1307072 (PMC4080523; doi:10.1289/ehp.1307072)
Supplement: (227 KB) PDF [file ehp.1307072.s001.pdf]

Environmental Health Perspectives doi:10.1289/ehp.1307072

Supplemental Material

The Influence of Declining Air Lead Levels on Blood Lead–Air Lead Slope Factors in Children

Jennifer Richmond-Bryant, Qingyu Meng, Allen Davis, Jonathan Cohen, Shou-En Lu, David Svendsgaard, James S. Brown, Lauren Tuttle, Heidi Hubbard, Joann Rice, Ellen Kirrane, Lisa C. Vinikoor-Imler, Dennis Kotchmar, Erin P. Hines, and Mary Ross

The choice of a ln-ln model formulation was evaluated using Monte Carlo simulations devised to provide a close representation of the ambient air lead (PbA) and blood lead (PbB) data held by the Research Data Center (RDC) for the merged National Health and Nutrition Examination Survey (NHANES) data set.

Simulation methods

To mimic the data structure of the merged PbB-PbA NHANES cohort, Equation [1] was used to simulate the original PbB data with simulated data for PbA, b_j, and $\varepsilon_{i,j}$ along with the β_0 and β_{PbA} estimates obtained from the fitted model for each NHANES survey and age group (defined in the Methods section, presented in Table 1, and described further in the Results and Discussion section). Specifically, the data for ln[PbA] were simulated based on a normal distribution with mean and variance close to the original data. This was validated by comparing multiple percentiles between the simulated distributions and the original ln[PbA] distributions. Random effects b_j were simulated from the normal distributions with zero mean and variance based on the location random effect reported in Table 1. Data for the random error $\varepsilon_{i,j}$ were simulated from either the normal distribution or the skewed normal distribution. If the goodness-of-fit test applied to the residuals from the fitted models in Table 1 supported the assumption that the random errors followed a normal distribution, data for $\varepsilon_{i,j}$ were simulated using a normal distribution with mean and variance equal to the sample mean and sample variance of the residuals. If not, a skewed normal distribution was applied with parameters determined by the mean, variance and skewness of the residual distribution. The choice of parameters was validated by comparing multiple percentiles between the simulated distributions and the original residual distributions. Data generation was repeated 1,000 times for each NHANES survey and age group.

Methods of model evaluation

The simulated data were fit to four models: ln-ln, ln-linear, linear-ln, and linear. Performance of these models was evaluated based on mean squared error (MSE) and percent of times (i.e., power) that the association H_0:$\beta_{PbA} = 0$ is rejected under $\alpha = 0.05$ (two-sided), based on 1000 simulation runs. MSE measures the closeness of the model predicted values and the observed values. Small MSE implies that the model predicted values and the observed values are close. Power was evaluated because it provides information on which model fitting is more powerful than the others in testing H_0: $\beta_{PbA} = 0$, and whether the result of β_{PbA} reported in Table 1 is robust in terms of evaluating the association between PbB and PbA among these four models.

Simulation results

Results are summarized in Supplementary Tables S1 and S2. A Monte Carlo approach was applied, and the summarized results represent the ensemble statistics across the 1,000 simulations. Results showed that the ln-ln and ln-linear models had similar MSE, and their MSE's were much smaller than those of the linear-ln and linear models, suggesting that the differences between the predicted ln[*PbB*] values of the ln-ln and ln-linear models and the observed ln[*PbB*] values were much smaller than the differences between the predicted PbB values from the linear-ln and linear models and the observed PbB values. However, we noted that the differences between the predicted PbB values based on the ln-ln and ln-linear models (after exponentiating the model predicted ln[*PbB*] values) and the observed PbBs (noted with a superscript a in Table S1) are close to, yet slightly larger than those of the linear-ln and linear models.

For the assessment of power, the performance of the ln-ln model is consistent with the significance (p-value) of the PbB-PbA association reported in Table 1 using the original data (see Table S2). Among the models, the ln-ln model has the highest power. It was also noted that when the PbB vs. PbA association is not significant in the original analysis (i.e., the analysis results reported in Table 1), none of the four models had sufficient power (<80%) to test such an association. When the PbB vs. PbA association was significant in the original analysis, the ln-ln and ln-linear models typically had sufficient power (>80% power) to test this association. In summary, these results suggest that the ln-ln model seemed valid and most appropriate for use in predicting slope factor, d[*PbB*]/d[*PbA*]. Results reported in Table 1 were robust against other choices of the model.

Table S1. MSE [mean (variance)] obtained for comparing PbB predictions for various model formulations while testing H_0: $\beta_{PbA} = 0$ of the ln-ln, ln-linear, linear-ln, and linear model formulations, based on 1000 simulation runs in each age group of the NHANES cohort.

Age group	ln-ln	ln-linear	ln-ln[a]	ln-linear[a]	linear-ln	linear-linear
**NHANES 9908**						
1-5 yr	0.158 (0.002)	0.157 (0.002)	1.998 (0.489)	1.990 (0.487)	1.652 (0.522)	1.648 (0.522)
6-11 yr	0.106 (0.000)	0.104 (0.000)	0.750 (0.061)	0.742 (0.060)	0.610 (0.064)	0.605 (0.066)
12-19 yr	0.156 (0.000)	0.154 (0.000)	0.463 (0.009)	0.459 (0.009)	0.391 (0.008)	0.387 (0.008)
20-59 yr	0.284 (0.001)	0.281 (0.001)	1.445 (0.069)	1.438 (0.068)	1.277 (0.071)	1.267 (0.070)
60+ yr	0.217 (0.001)	0.217 (0.001)	2.284 (0.220)	2.284 (0.220)	2.099 (0.236)	2.098 (0.237)
**NHANES III**						
1-5 yr	0.351 (0.003)	0.350 (0.003)	19.419 (24.784)	19.391 (24.739)	16.250 (25.453)	16.225 (25.469)
6-11 yr	0.103 (0.001)	0.103 (0.001)	6.452 (8.589)	6.426 (8.405)	4.148 (8.413)	4.149 (8.481)
12-19 yr	0.113 (0.002)	0.111 (0.001)	2.221 (0.831)	2.195 (0.816)	1.556 (0.960)	1.548 (0.963)
20-59 yr	0.371 (0.002)	0.367 (0.002)	10.666 (3.068)	10.637 (3.043)	9.246 (3.308)	9.187 (3.292)
60+ yr	0.331 (0.003)	0.330 (0.003)	14.077 (7.906)	14.048 (7.921)	12.551 (7.872)	12.527 (7.946)

[a]MSE of the predicted PbB values (i.e., after exponentiating the predicted ln[PbB] values) from the ln-ln and ln-linear models and the observed PbB values. In contrast, other columns present MSE for the predicted ln[PbB].

Table S2. Evaluation of power testing for H_0: $\beta_{PbA} = 0$ of the ln-ln, ln-linear, linear-ln, and linear model formulations, based on 1000 simulation runs in each age group of the NHANES cohort and $\alpha = 0.05$ (two-sided).

**Age group**	**ln-ln**	**ln-linear**	**linear-ln**	**linear**	**Estimated β_{PbA}**
**NHANES 9908**					
1-5 yr	0.468	0.282	0.397	0.256	0.076
6-11 yr	0.981	0.853	0.954	0.783	0.155**
12-19 yr	0.995	0.908	0.991	0.842	0.120#
20-59 yr	0.975	0.781	0.954	0.701	0.089**
60+ yr	0.002	0.006	0.003	0.011	-0.023
**NHANES III**					
1-5 yr	0.911	0.843	0.834	0.721	0.140*
6-11 yr	0.662	0.544	0.565	0.448	0.154
12-19 yr	0.991	0.951	0.973	0.898	0.225#
20-59 yr	0.999	0.996	0.999	0.993	0.168#
60+ yr	0.873	0.761	0.794	0.663	0.115**

*$0.01 < p \leq 0.05$; **$0.001 < p \leq 0.01$; #$p \leq 0.001$
